# Evaluation of an online, real-time, soft-photon ionisation time-of-flight mass spectrometer for mainstream tobacco smoke analysis

**DOI:** 10.1186/s13065-019-0654-z

**Published:** 2019-12-21

**Authors:** Jenni Hawke, Graham Errington, Matthias Bente von Frowein

**Affiliations:** 1 British American Tobacco, Group R&D Centre, Southampton, SO15 8TL UK; 2 Photonion GmbH, Hagenower Str. 73, 19061 Schwerin, Germany

**Keywords:** SPI-TOF, Mainstream cigarette smoke, Acetaldehyde, Acetone, 1,3-Butadiene, 2-Butanone, Benzene, Isoprene, Toluene, Puff by puff

## Abstract

Mainstream tobacco smoke is a complex and dynamic aerosol, consisting of particulate and vapour phases. Most approaches to determine mainstream smoke toxicant yields are based on offline techniques that limit the opportunity to observe in real time the processes leading to smoke formation. The recent development of online real-time analytical methods offers many advantages over traditional techniques. Here we report the LM2X-TOFMS (Borgwaldt GmbH, Germany), a commercial instrument that couples a linear smoking engine with a time-of-flight mass spectrometer for real-time per-puff measurement of the vapour phase of mainstream cigarette smoke. Total cigarette and puff-by-puff (μg/puff) yields were evaluated, in line with International Council of Harmonisation recommendations, for seven smoke toxicants: acetaldehyde, acetone, 1,3-butadiene, 2-butanone, benzene, isoprene and toluene. Measurements were unaffected by small system changes including replacing the sampling capillary or time of day (all *P *> 0.05), indicating that the LM2X-TOFMS is rugged. Control charts showed that the system has good stability and control. Analysis of certified gas mixtures of six concentrations of each analyte showed a highly linear response for all seven analytes (R^2^ = 0.9922–0.9999). In terms of repeatability, the lowest variation was observed for isoprene with a coefficient of variation (CV) of < 6% for each concentration. Acetaldehyde showed the highest CV, increasing from 8.0 to 26.6% with decreasing gas concentration. Accuracy was analysed in terms of relative error, which was ± 16% for six of the analytes; however, the relative error for acetaldehyde was (− 36.2%), probably due to its low ionisation efficiency under the instrument’s vacuum ultraviolet lamp. Three cigarette products (reference and commercial) with different ISO tar levels were analysed by the LM2X-TOFMS puff by puff under ISO regulatory smoking conditions. The relative standard deviation based on average yield per cigarette for each analyte in each product (summed puffs per product, n = 30) ranged from ≤ 9.3 to ≤ 16.2%. Measurements were consistent with published data per cigarette. In conclusion, the LM2X-TOFMS is suitable for determining the vapour-phase yields of seven analytes on a real-time, puff-by-puff basis, and can be utilised for both fast screening (qualitative) and quantitative measurements of mainstream cigarette smoke.

## Introduction

Mainstream smoke is a complex and dynamic aerosol, consisting of particulate and vapour phases generated by a combination of combustion, pyrolysis and distillation. More than 6500 unique chemical components, including many toxicants, have been identified in tobacco smoke [1] and, as analytical techniques continue to advance, this number is likely to increase.

Measurement and reporting of toxicant emissions from cigarettes is already mandated in Canada [2]. In the United States, the Food and Drug Administration (FDA) has published a list of 93 harmful and potentially harmful constituents (HPHCs) in tobacco products and tobacco smoke [3] and issued draft guidance on the reporting of 18 of these HPHCs [4]. Similarly, the World Health Organisation Study Group on Tobacco Product Regulation, which is working towards a scientific basis for tobacco product regulation [5], has proposed the measurement and reporting of selected smoke toxicants and some compounds in cigarette filler blends [6].

The main approach to the composition testing of cigarette smoke includes standardised machine-smoking protocols (e.g., ISO 3308 [7] and Health Canada Intense (HCI) [2]), coupled with collection of particulate phase smoke on Cambridge filter pads and offline analysis by various methods including gas chromatography (GC), high-performance liquid chromatography (HPLC) and mass spectrometry (MS). Using such methods, numerous studies have reported toxicant yields in mainstream smoke (e.g., [8–12]). More recently, volatile organic compounds have been quantified in cigarette smoke via the collection of vapour-phase smoke samples into gas sampling bags [13, 14].

Many MS ionisation techniques cause significant fragmentation of the chemical substances present, leading to complex spectra and corresponding difficulty in the deconvolution of multiple spectra, leading to a requirement for additional (e.g., chromatographic) separation. Online measurement techniques that facilitate real-time quantifiable yield measurements could provide many advantages over established techniques, including insight into the processes that lead to smoke and toxicant formation.

In the past 10 years, soft photoionization (SPI) MS techniques have been advancing toward the online analysis of complex mixtures such as tobacco smoke (e.g., see Refs. [15–19]). The low energy of SPI (7.9 eV to 11.6 eV) relative to electron impact ionisation (70 eV) results in almost no fragmentation of the chemical species and therefore much simpler spectra for deconvolution. In initial studies on tobacco, Adam et al. [15] showed that, coupled with statistical analysis, SPI time-of-flight MS (TOFMS) could differentiate between mainstream smoke samples generated from the three major types of tobacco: Burley, Virginia and Oriental. Tobacco samples were pyrolysed at 800 °C in a nitrogen atmosphere, and the resulting aerosol was passed directly to the ion volume of the TOFMS. Principal component analysis and linear discriminant analysis were used to differentiate the spectra of the three samples, each of which contained signals from more than 70 species between *m*/*z* 5 and *m*/*z* 170. The same research group has also coupled resonance-enhanced multiphoton ionisation (REMPI) and SPI with TOFMS to achieve the online analysis of cigarette mainstream smoke [18]. In this case, an optimised smoking machine was connected directly to the REMPI/SPI-TOFMS instrument, enabling puff-by-puff resolved measurements of chemical constituents of mainstream cigarette smoke.

Subsequent studies have characterised and compared the puff-by-puff resolved and total yields of cigarette mainstream smoke [16], as well as puff-by-puff measurement of selected toxicants, including acetaldehyde, butadiene, acetone, isoprene, benzene and toluene [20]. The puff-resolved smoke profiles demonstrate that the yields of cigarette smoke constituents can differ significantly between puffs. For many smoke constituents, the concentration is high in the lighting puff, lower in puff 2 and then increases gradually thereafter, mainly because more tobacco/tar mass is burned in later puffs due to tar deposition in the tobacco rod from earlier puffs. Thus, the practicality of SPI to investigate organic compounds in complex gas mixtures in real time has been clearly established. Furthermore, puff-by-puff analysis facilitated by SPI-TOFMS should aid our understanding of the formation and decomposition reactions that occur when a cigarette is smoked [21, 22] and thus guide targeted reduction strategies for specific toxicants or groups of toxicants in the smoke.

The aim of the present study was therefore to test and evaluate the performance of the LM2X-TOFMS instrument—a commercial system developed by Borgwaldt GmbH (Germany) for the online analysis of mainstream tobacco smoke. The LM2X-TOFMS was used to quantify the total and puff-by-puff yields of seven vapour-phase smoke constituents (acetaldehyde, acetone, benzene, 1,3-butadiene, 2-butanone, isoprene and toluene), six of which are included in the FDA’s list of HPHCs in tobacco and smoke [3]. Through a series of measurements of certified gas mixtures and cigarette smoke generated under ISO regulatory puffing regimes [7], the LM2X-TOFMS has been evaluated for ruggedness, stability, linearity, repeatability/reproducibility and accuracy in line with International Council of Harmonisation recommendations [23].

### The LM2X-TOFMS system

The LM2X-TOFMS system has been developed as a commercial system by Borgwaldt GmbH (Germany) and Photonion GmbH (Germany) for a range of industrial and research applications, including the online analysis of cigarette smoke.

**Fig. 1:**
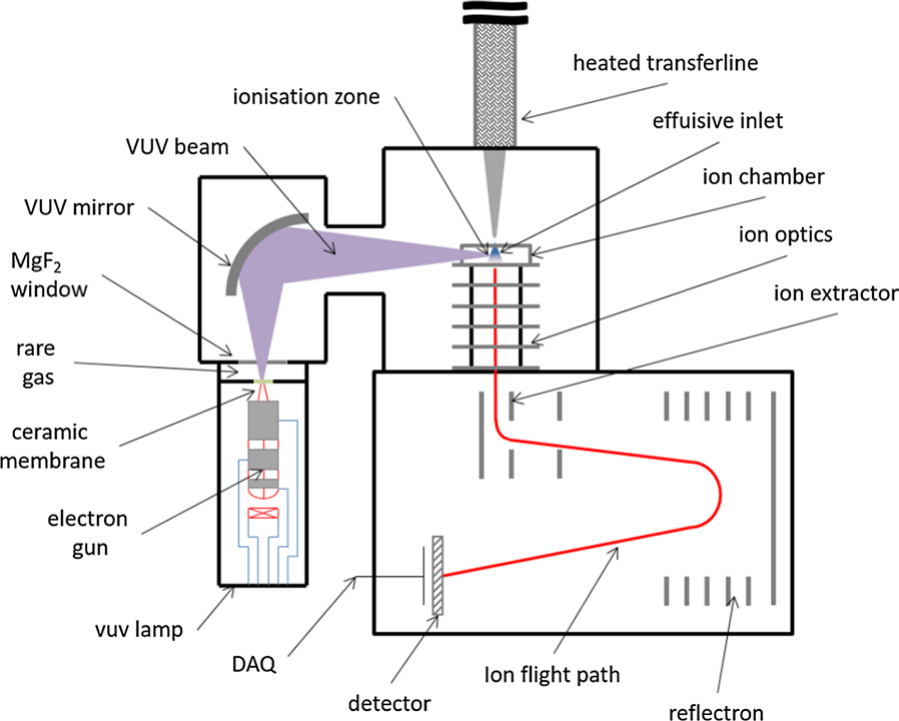
Schematic of the time-of-flight mass spectrometer

The LM2X-TOFMS comprises a linear smoking engine coupled to an orthogonal TOF mass spectrometer, which facilitates real-time, per-puff analysis of the vapour phase of mainstream cigarette smoke. The smoking machine consists of a cigarette holder connected to a valve, through which are drawn fixed “puffs” of smoke from the burning cigarette. In turn, the valve is connected by a heated transfer line, containing a deactivated fused silica capillary (o.d., 350 μm; i.d., 180 μm; length, ~ 3.3 m), to the orthogonal TOF mass spectrometer, enabling a subsample of each puff to be analysed (Fig. 1). Full details of the TOF mass spectrometer are given in [24].

A vacuum ultraviolet (VUV, wavelength 126 nm) lamp is used as the light source for SPI. This ionisation technique causes virtually no fragmentation of the chemical species present in the sample and enables substances in the complex mainstream smoke sample to be measured directly, while background gases such as O_2_, N_2_ and CO_2_, which have ionisation potentials greater than 9.8 eV, are not ionised and do not overload the detector. VUV photons are produced by excitation of inert argon gas with an electron beam. A more detailed description is given in Mühlberger et al. [25]. The VUV photons are directed from the lamp by a mirror to the target in the ion volume, which is located at the bottom of the sample capillary. Molecules present in the mainstream smoke subsample are hit by the photons, becoming positive ions. As in a standard TOF instrument, the TOF mass analyser measures the time that it takes for these ions to ‘fly’ from one side of the drift tube to the other and hit the detector; the flight time is proportional to the mass-to-charge (*m*/*z*) ratio (Fig. 1).

The manufacturer’s specifications of the LM2X-TOFMS are given in Table 1. A full mass spectrum (*m*/*z* vs intensity) is produced for each sample. At present, the LM2X-TOFMS is performance-optimised for the analysis of seven species: three carbonyls, acetaldehyde (*m/z* 44), acetone (58) and 2-butanone (72); two aromatics, benzene (78) and toluene (92); and two alkenes, 1,3-butadiene (54) and isoprene (68). The concentration of each analyte is determined relative to the signal for toluene, which has been established as the calibration gas (100 ppm in N_2_). The LM2X-TOFMS software automatically analyses and calculates smoke yield data, which are output as total mass (yield) per cigarette or puff-by-puff mass (yield). The internal algorithm is based on ISO puffing conditions (35 mL over 2 s, every 60 s [7]) and the ideal gas law equation.

**Table 1:** Specifications of the LM2X-TOFMS

Mass range	1–600 Th (m/z)
Dynamic range	1E6
Resolution	700 (m/Δm, FWHM)
Mass accuracy	100 ppm
Gas flow into ion volume	~ 0.7 mL/min
Calibration gas	100 ppm toluene in N_2_

## Experimental

### Materials and smoking conditions

A reference cigarette (3R4F, Center for Tobacco Reference Products, University of Kentucky, USA) was used for ruggedness testing. A further reference cigarette (CORESTA monitor, CM6), 3R4F and a commercial cigarette (DW) were used to test repeatability and reproducibility across a range of yields. All cigarettes were standard king-sized products of 83 mm length (including a 27 mm cellulose acetate filter) and 27 mm circumference. The product data from ISO testing were as follows: 3R4F, 9.4 mg/cig nicotine-free dry particulate matter (NFDPM) and 0.7 mg/cig nicotine; CM6, 14 mg/cig NFDPM and 1.4 mg/cig nicotine; commercial cigarette (DW), 1.9 mg/cig NFDPM and 0.2 mg/cig nicotine. All cigarette samples were conditioned for at least 48 h but no more than 10 days under ISO conditions [26]: temperature, 22 ± 1 °C; relative humidity, 60 ± 3%. Any cigarettes with visible defects were discarded. After conditioning, cigarettes were smoked to the butt length (i.e., tipping paper length plus 3 mm) by using ISO smoking regime parameters: 35-mL volume, bell-shaped puff, duration 2 s, interval 60 s (no ventilation blocking) [7].

### Gas mixtures: source and specifications

Certified standard gas bottles containing six different concentrations of the seven quantifiable constituents were purchased from Air Products (Surrey, UK) (see Table 3). Each constituent had its own concentration range, established from reported smoke yields (μg/puff), to ensure that the full concentration range possible from mainstream tobacco smoke would be represented under the ISO regulatory smoking regime [7] used, and would extend to a more intense HCI [2] regulatory regime. The highest gas concentration was higher than the yields reported for an HCI data set [9, 27]. The smoke yield data were converted from μg/puff to ppm assuming ideal gas conditions for all parameters and a temperature of 22 °C.

### Ruggedness measurements

Ruggedness was tested by making deliberate changes to parameters including capillary length, ferrule (used or new), day and time of day (morning or afternoon). Initially, 30 test runs (where a run indicates measurement of all puffs for a single cigarette for all seven analytes) were completed over 3 days (5 morning and 5 afternoon runs per day). In further tests of day-to-day variability, 30 runs were conducted over 5 days with 3, 6, 4, 5 and 12 runs on consecutive days. Each run comprised seven puffs of a 3R4F cigarette under ISO smoking conditions [7]. Only one operator performed all measurements. Leak, puff volume and air flow checks were performed on the LM2X-TOFMS prior to cigarette sample measurement.

### Gas bag measurements (linearity, accuracy and repeatability)

The gas mixtures were analysed via 2-L Tedlar gas bags, which were filled and emptied three times with the certified calibrant to prevent losses due to absorption before analysis. Separate gas bags were used for each gas mixture. Gas bags filled with only nitrogen were analysed as blanks. Consistent with ISO puffing parameters [7], the smoke engine drew 35-mL samples (“puffs”) from the gas bag for analysis. Measurements were performed over 3 days with 10 puffs of each gas mixture analysed twice in a random order every morning and afternoon (n = 120 puffs per gas mixture). Leak and puff volume checks were performed on the LM2X-TOFMS prior to cigarette sample measurement.

### Cigarette analysis

Cigarettes were smoked under ISO conditions [7] over 5 days with 18 runs per day. Each run involved up to eight puffs of one cigarette. Smoke runs were randomised in terms of product and one operator performed all measurements. Clearing puffs were also performed after each run to prevent residual effects from deposition. The analyte yield per cigarette was determined by smoking each product to the marked butt length. Measurements were performed according to ISO 4387:2000 [28], where butt length is specified as the tipping paper length plus 3 mm. Thirty cigarettes per product were analysed.

### Data analysis

Data were analysed in Excel (Microsoft, Redmond, WA, USA). Yields of the seven analytes were reported as mean ± SD. Relative error was determined as (concentration measured − concentration expected)/concentration expected, and was reported as a percentage. Ruggedness was tested by one-way analysis of variance. Linearity was tested by linear regression of the calculated response versus the measured response.

**Fig. 2:**
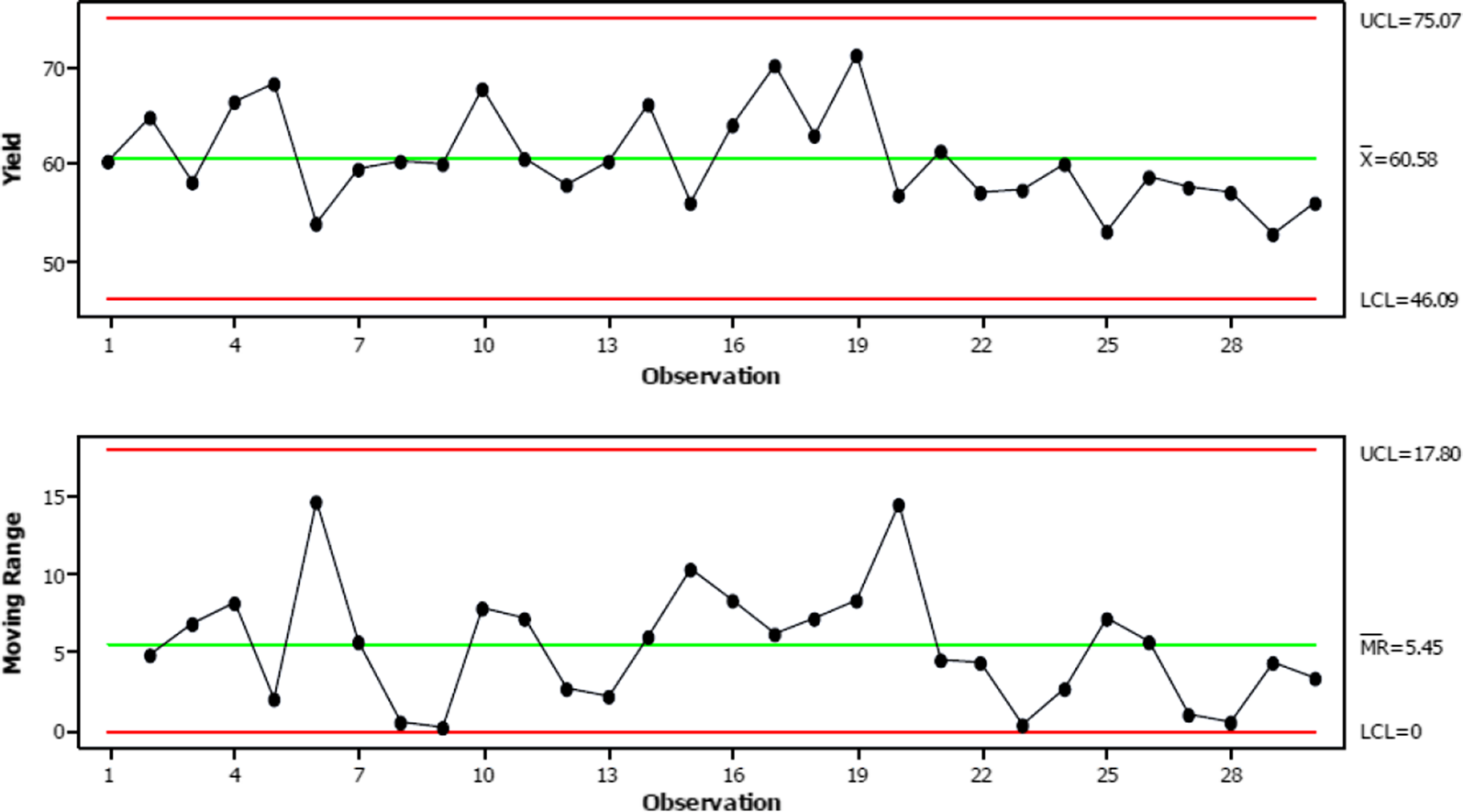
Control chart for toluene as (μg/puff), showing overall variability in repeat measurements. Data were recorded over 5 days with 3, 6, 4, 5 and 12 repeat measurements per run. Top, individual measurements (n = 30). Bottom, moving range

The analysis of variance (ANOVA) General Linear Model in Minitab (version 17, Minitab Inc, State College PA, USA) was used to calculate the repeatability (r) and reproducibility (R) of the gas bag measurements for each analyte. The variables were puff number, day and time of day, and the mean squared error (Sr) per variable was reported. To allow for 99% coverage under the assumption of normally distributed data, Sr was multiplied by 2.8 to determine r, as recommended in ISO 5725-2 [29]. The stability of the system was assessed by plotting individual and moving range control charts in Minitab (see Fig. 2 for the toluene chart).

## Results and discussion

### Ruggedness

Experiments were conducted to assess the effects of small but deliberate changes in operational factors, such as reducing the length of the capillary between the cigarette valve and the ion volume, installing a new ferrule, and the day and time of day of measurement. In total, 30 Kentucky 3R4F cigarettes were smoked (10 per day for 3 days) with changes to the capillary and ferrule made each morning and afternoon of each day in a controlled manner (Additional file 1: Table S1). Overall, the mean ± SD (range) yields per cigarette (n = 30) ranged from 27.3 ± 3.3 (18.5–32.5) μg/cig for 1,3-butadiene to 387.4 ± 54.2 (293.0–508.0) μg/cig for acetaldehyde (Table 2).

**Table 2:** Ruggedness *P* values according to one-way ANOVA by constituent

Analyte	Mean ± SD, μg/cig (n = 30)	LOQ^a^, μg	Capillary (new vs used)	Ferrule (new vs used)	Day	Time (a.m. vs p.m.)	Treatment
Acetaldehyde	387.4 ± 54.2	17	0.290	0.156	0.074	0.808	0.704
1,3-Butadiene	27.3 ± 3.3	3.5	0.676	0.725	0.084	0.518	0.798
Acetone	174.7 ± 16.6	0.9	0.980	0.83	0.014^b^	0.830	0.796
Isoprene	270.9 ± 36.9	2.1	0.826	0.96	< 0.001^b^	0.809	0.878
2-Butanone	61.8 ± 5.5	0.9	0.828	0.584	0.138	0.774	0.670
Benzene	29.6 ± 3.	0.8	0.286	0.725	0.027^b^	0.729	0.386
Toluene	61.8 ± 6.1	2.8	0.500	0.635	0.003^b^	0.708	0.818

^a^Limit of quantification for a 35-mL 2-s puff of a standard gas sample with an assumed signal-to-noise ratio of 10

^b^Significant at *P *< 0.05

By ANOVA, no statistically significant differences (*P *≥ 0.05) were found for capillary, ferrule or time of day (a.m. vs p.m.) for any of the seven constituents (Table 2). In addition, “treatment”, defined as a combination of the small changes (e.g., a measurement with a new capillary and ferrule performed on day 1 in the morning), did not lead to significant differences in the data. Thus, changing the capillary, ferrule or time of day when measurements are performed does not affect yield measurements for the LM2X-TOFMS. However, a significant difference (*P *< 0.05) was seen in day-to-day variation for four of the seven constituents (acetone, isoprene, benzene and toluene). As a result, further measurements to analyse the day-to-day variation were carried out.

### Day-to-day variability

A further 30 repeat runs were carried out over 5 days with a different number of runs per day (3, 6, 4, 5 and 12) to specifically analyse the day-to-day variation. In this test, each 3R4F cigarette sample was removed individually from the conditioning room immediately before analysis. One-way ANOVA of the 30 measurements showed that day was not a significant factor for any of the seven analytes (acetaldehyde, *P *= 0.063; 1,3-butadiene, *P *= 0.603; acetone, *P *= 0.510; isoprene, *P *= 0.576; 2-butanone, *P *= 0.639; benzene, *P *= 0.597; toluene, *P *= 0.169).

The raw data (reported as μg/puff derived from the instrument algorithm, post toluene calibration) from the repeat measurements (n = 30) were analysed in Minitab to produce control charts for each analyte to determine whether the LM2X-TOFMS operates in a controlled and stable manner. Apart from toluene, all data points on the individual charts lay within the control limits (data not shown). For toluene, one point of the moving range chart was just outside the upper control limit (UCL). The other 11 measurements on that day showed similar yields and group around the calculated mean, suggesting that the first point was an outlier. In the control chart of overall variability across the 5 days (Fig. 2), all data points were within the control limits. As shown in Fig. 2, there was a gradual shift in mean because the last nine points were below the mean line. This deviation was noted during data analysis; if observed during operation, it would trigger further investigation as per the rule set for Shewhart control charts [30].

Taken together, the individual control charts for all analytes confirm that, although there is day-to-day variation, some of which might be due to cigarette variation (typically 4–10%; [27]), the overall analytical process of the LM2X-TOFMS shows good stability and control.

### Linearity

The linearity of the LM2X-TOFMS was tested by analysing gas mixtures with certified concentrations of the seven analytes. During this analysis, the temperature used in the ideal gas law equation by the internal algorithm was amended from the heated gas valve temperature (150 °C) to room temperature (22 °C) as the puff volume (35 mL) was sampled at room temperature. The mean values of the measured response (n = 120 puffs per mixture) are presented in Table 3.

**Table 3:** Gas mixture analysis for linearity check

Analyte (m/z)	Gas bottle concentration (ppm)	Calculated response (μg/puff)	Measured response, mean ± SD (μg/puff)
Acetaldehyde (44)	199.5	12.7	8.1 ± 2.1
	499.5	31.8	20.4 ± 3.7
	747	47.5	0.2 ± 0.3^a^
	998	63.5	41.4 ± 4.6
	1500	95.4	61.0 ± 5.4
	2000	127.2	81.6 ± 7.0
Acetone (58)	50.83	4.3	3.6 ± 0.4
	99.76	8.5	7.1 ± 0.7
	199.2	16.9	14.2 ± 0.9
	297.4	25.3	21.4 ± 1.2
	399.3	33.9	28.5 ± 1.3
	499.9	42.5	35.6 ± 1.9
1,3-Butadiene (54)	9.7	0.8	0.8 ± 0.2
	19.5	1.5	1.8 ± 0.2
	38	3.0	3.1 ± 0.5
	60.4	4.7	5.3 ± 0.4
	76.6	6.0	6.2 ± 0.6
	96.3	7.5	8.6 ± 0.6
2-Butanone (72)	21.17	2.2	2.0 ± 0.3
	29.68	3.1	2.9 ± 0.4
	39.85	4.1	3.8 ± 0.4
	61.33	6.4	5.9 ± 0.5
	80.01	8.3	7.6 ± 0.5
	99.24	10.3	9.5 ± 0.6
Benzene (78)	10	1.1	1.1 ± 0.2
	20.07	2.3	2.2 ± 0.2
	40.15	4.5	4.3 ± 0.3
	59.83	6.8	6.6 ± 0.4
	71.06	8.0	7.6 ± 0.4
	78.31	8.8	8.5 ± 0.5
Isoprene (68)	98.6	9.7	10.9 ± 0.6
	199.3	19.5	22.4 ± 1.0
	299.6	29.4	33.6 ± 1.1
	400.4	39.2	44.8 ± 2.6
	501	49.1	56.3 ± 1.7
	594.6	58.3	66.7 ± 2.2
Toluene (92)	15.21	2.0	2.0 ± 0.2
	29.88	4.0	4.0 ± 0.3
	39.87	5.3	5.4 ± 0.4
	60.32	8.0	8.0 ± 0.4
	80.46	10.7	10.7 ± 0.5
	99.5	13.2	13.2 ± 0.6

^a^Due to an error by the supplier, acetaldehyde had been omitted from the corresponding gas bottle and this data point was therefore excluded from the analysis

**Fig. 3:**
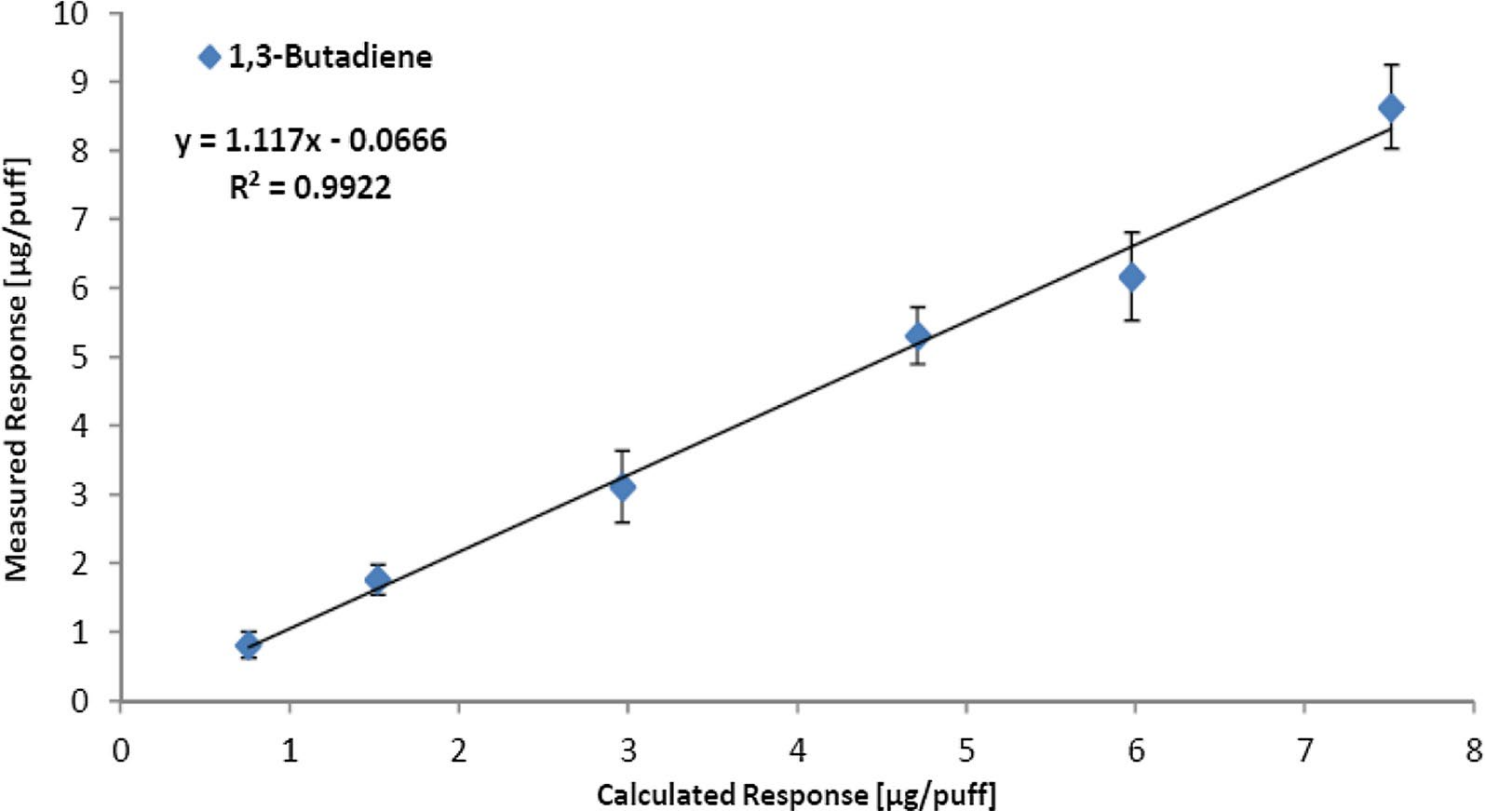
Linear regression of the calculated and measured yields of 1,3-butadiene, illustrating the linearity of the LM2X-TOFMS

To establish linearity, the mean values were plotted against the calculated response for each analyte, a linear fit was chosen, and the *R*^2^ values were calculated for each analyte. As an example, Fig. 3 shows that the response for 1,3-butadiene was highly linear (*R*^2^ = 0.9922).

The response for acetaldehyde, acetone, 2-butanone, benzene, isoprene and toluene was also highly linear with *R*^2^ values of 0.9999, 0.9999, 0.9995, 0.9996, 1.000 and 0.9999, respectively (Additional file 1: Figure S1). Thus, all seven analytes demonstrated excellent linearity across all gas concentrations tested.

### Accuracy

Accuracy was evaluated in terms of the relative error, which was determined for the gas bag measurements (Table 4). The errors for acetaldehyde, acetone and isoprene were consistent across the minimum, maximum and mean values. These errors are therefore likely to be systematic and could be modified by applying a correction factor to the raw data. Systematic errors were also observed for 2-butanone and benzene, but because the values were small (< 10%), there would be no need to correct the raw data. Non-systematic errors were observed for 1,3-butadiene and toluene, where the biggest variation occurred at higher concentrations. However, the error for toluene was small (< 10%).

**Table 4:** Percentage relative error for the gas bag measurements

Analyte	Mean^a^	Minimum	Maximum
Acetaldehyde	− 35.7	− 36.2	− 34.8
Acetone	− 16.0	− 16.3	− 15.2
1,3-Butadiene	9.7	3.2	16.1
2-Butanone	− 8.1	− 10.8	− 5.6
Benzene	− 3.8	− 5.2	− 2.0
Isoprene	14.3	12.9	14.7
Toluene	0.35	− 0.36	1.78

^a^Determined from 120 puffs across six concentrations for all analytes except acetaldehyde (100 puffs across five concentrations)

### Repeatability and reproducibility

Repeatability (r) is the maximum difference expected between two sample measurements within a run, whereas reproducibility (R) is the maximum difference between two samples measured either in different laboratories by different operators or simply by different operators. Because this was the first commercial LM2X-TOFMS instrument, it was not possible to measure R in the former way; the present data were also obtained by one operator. Thus, reproducibility in this study indicates the maximum difference observed between two measurements, performed on different days at different times (morning or afternoon). The repeatability and reproducibility of the gas bag measurements are presented in Table 5.

**Table 5:** R and r values for the gas bag measurements

Analyte	Gas bottle conc., ppm	Measured mean ± SD yield, µg/puff	R	r	CV(R), %	CV(r), %
Acetaldehyde	199.5	8.1 ± 2.1	8.0	6.0	35.1	26.6
	499.5	20.4 ± 3.7	10.2	10.5	17.6	18.4
	998	41.4 ± 4.6	20.3	13.0	17.5	11.2
	1500	61.0 ± 5.4	53.4	14.2	31.2	8.3
	2000	81.6 ± 7.0	73.3	18.3	32.1	8.0
Acetone	50.83	3.6 ± 0.4	2.5	1.2	24.6	12.2
	99.76	7.1 ± 0.7	4.4	1.8	22.3	9.1
	199.2	14.2 ± 0.9	6.4	2.4	16.1	6.0
	297.4	21.4 ± 1.2	4.5	3.3	7.4	5.4
	399.3	28.5 ± 1.3	9.5	3.8	12.0	4.7
	499.9	35.6 ± 1.9	19.8	5.1	19.9	5.1
1,3-Butadiene	9.7	0.8 ± 0.2	1.1	0.5	49.4	22.9
	19.5	1.8 ± 0.2	1.6	0.6	31.8	12.2
	38	3.1 ± 0.5	3.6	1.4	41.1	16.1
	60.4	5.3 ± 0.4	1.9	1.2	12.6	8.0
	76.6	6.2 ± 0.6	7.0	1.6	40.7	9.1
	96.3	8.6 ± 0.6	6.3	1.6	26.2	6.6
2-Butanone	21.17	2.0 ± 0.3	0.9	0.8	16.0	15.3
	29.68	2.9 ± 0.4	1.4	1.0	16.9	12.2
	39.85	3.8 ± 0.4	2.0	1.1	18.4	10.2
	61.33	5.9 ± 0.5	2.1	1.5	12.3	8.8
	80.01	7.6 ± 0.5	3.0	1.5	14.3	7.2
	99.24	9.5 ± 0.6	4.0	1.6	15.0	6.0
Benzene	10	1.1 ± 0.2	0.9	0.5	30.4	15.4
	20.07	2.2 ± 0.2	1.5	0.7	23.4	10.5
	40.15	4.3 ± 0.3	2.2	0.9	18.4	7.8
	59.83	6.6 ± 0.4	2.1	1.0	11.3	5.5
	71.06	7.6 ± 0.4	2.3	1.2	10.9	5.4
	78.31	8.5 ± 0.5	4.3	1.3	18.0	5.4
Isoprene	98.6	10.9 ± 0.6	2.3	1.8	7.4	5.8
	199.3	22.4 ± 1.0	7.2	2.6	11.5	4.1
	299.6	33.6 ± 1.1	8.0	3.1	8.5	3.3
	400.4	44.8 ± 2.6	11.9	7.4	9.5	5.9
	501	56.3 ± 1.7	12.7	4.8	8.1	3.0
	594.6	66.7 ± 2.2	25.1	5.6	13.4	3.0
Toluene	15.21	2.0 ± 0.2	0.8	0.7	14.8	12.1
	29.88	4.0 ± 0.3	2.4	0.9	21.6	8.1
	39.87	5.4 ± 0.4	1.8	1.0	11.7	6.8
	60.32	8.0 ± 0.4	1.8	1.2	7.9	5.5
	80.46	10.7 ± 0.5	2.0	1.5	6.7	5.1
	99.5	13.2 ± 0.6	5.9	1.6	16.0	4.4

As expected, R was larger than r for all analytes at all six gas concentrations except for one concentration of acetaldehyde (499.5 ppm; Table 5). As a general principle of process control, a coefficient of variation (CV; or relative standard deviation, RSD) of less than 10% would be considered acceptable [31]; however, the mean value should also be considered because the CV may be high at very low concentrations and low at very high concentrations owing to the Horwitz trumpet effect [32]. Indeed, the biggest variations were observed for lower gas concentrations.

The smallest variation in repeatability (r) was observed for isoprene, for which all six gas concentrations demonstrated a CV of less than 6%. The second smallest variation was observed for toluene: for which the CV was less than 9% except at the lowest concentration (15.21 ppm) which had a CV of 12.1%. The largest variation was observed for acetaldehyde, which increased from 8.0% for the highest concentration (2000 ppm) to 26.6% for the lowest concentration (199.5 ppm).

The data provide limits for future reference. For example, in the case of two isoprene measurements performed on the same day at a yield of 66.7 μg/puff, the repeatability should be within 3.0% or 2.0 μg/puff. If the measurements were performed on different days (reproducibility), then the difference should be within 13.4% or 8.9 μg/puff.

Repeatability, r, was also assessed on an inter-day (between days) and intra-day (within day) basis (Table 6). Day 1 data were used for intra-day results as this was the 1st day that the gas bags were used (no sample carry over). Data from all 3 days were used to calculate the inter-day CV.

**Table 6:** Coefficient of variation for inter- and intra-day analysis

Analyte	Gas standard range	Intra-day CV, %		Inter-day CV, %	
		Mean	Range	Mean, %	Range, %
Acetaldehyde	199.5–2000	14.3	8.4–25.2	14.5	8.6–26.1
Acetone	50.8–499.9	7.6	3.7–14.5	7.2	4.7–12.1
1,3-Butadiene	9.7–96.3	12.8	6.9–26.9	13.0	7.0–23.6
2-Butanone	21.2–99.2	9.9	7.0–14.8	9.9	6.2–14.9
Benzene	10–78.31	9.1	5.2–18.1	8.6	5.4–15.8
Isoprene	98.6–594.6	3.7	2.5–6.5	4.2	3.1–5.7
Toluene	15.2–99.5	7.0	4.4–12.8	7.0	4.8–12.0

Intra-day CV was calculated from data obtained on day 1; inter-day CV was calculated from data obtained on days 1–3

### Stability

**Fig. 4:**
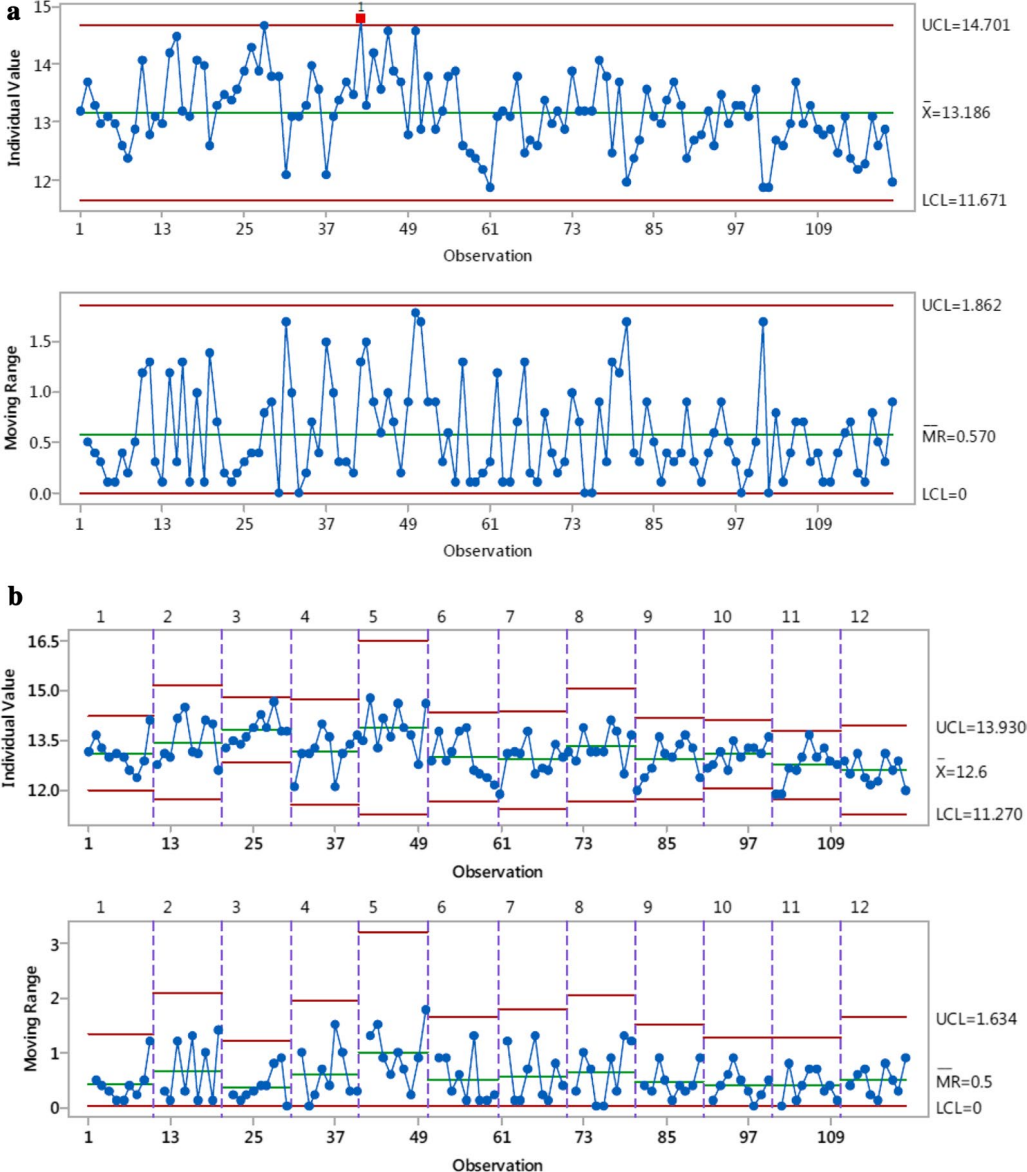
Individual moving range control charts for toluene yields (μg/puff) from the highest gas concentration (99.5 ppm). **a** Top, individual measurements for all data points (n = 120). Bottom, difference between two consecutive data points (moving range). **b** Variability in repeats per analytical run (n = 10). The charts in **a** were broken down into 12 sections with their own upper and lower control limits as indicated by the dotted lines. Top, individual measurements; bottom, difference between two consecutive data points

The stability of the system towards each analyte was further assessed on a per-puff basis by constructing individual moving range control charts. For a system to be deemed stable, the points in the charts should lie within the upper (UCL) and lower (LCL) control limits. This range should also reflect fitness for measurement. Using toluene as an example (Fig. 4), 119 of the 120 data points were within the control limits for both the individual measurements and moving range charts. Only one of the individual measurements lay just outside the UCL (Fig. 4a, top). Because up to 1 point in 25 can be outside these limits (Shewhart’s criterion [30]), the analytical process for toluene is considered stable and in control. When the variability in repeat measurements within a single analytical run was considered (Fig. 4b), three points in the moving range chart (bottom) were just outside the UCL; however, these data indicate the difference between two individual measurements that were within the UCL (top).

**Fig. 5:**
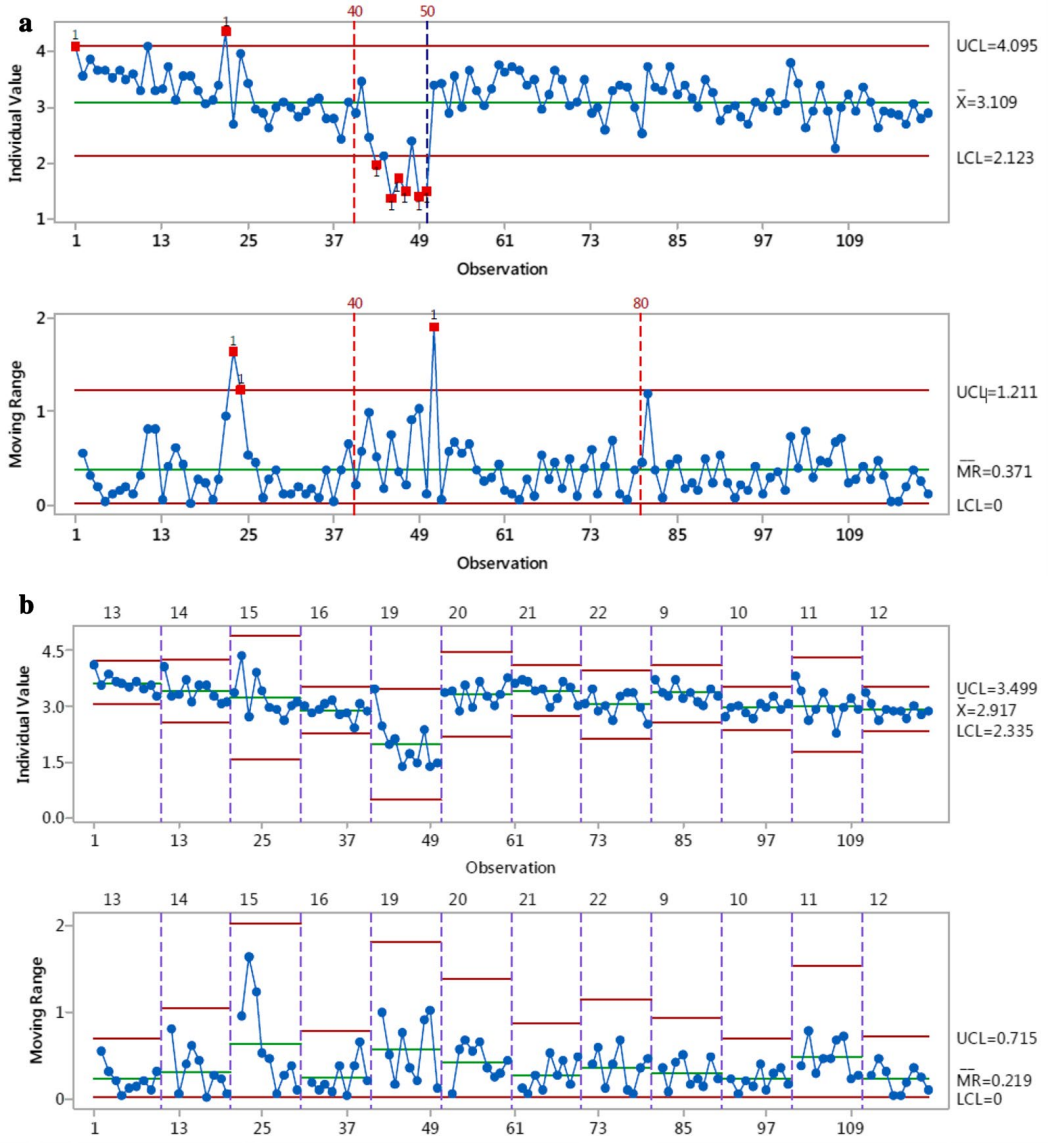
Individual moving range control charts for 1,3-butadiene yields (μg/puff) from the highest gas concentration (99.5 ppm). **a** Top, individual measurements for all data points (n = 120). Bottom, difference between two consecutive data points (moving range). **b** Variability in repeats per analytical run (n = 10). The charts in **a** were broken down into 12 sections with their own upper and lower control limits as indicated by the dotted lines. Top, individual measurements; bottom, difference between two consecutive data points

Regarding the other analytes, only 2 of the 29 control charts had data lying outside Shewhart’s criterion for statistical control: one for isoprene measurements of the 594.6 ppm gas concentration; and one for 1,3-butadiene measurements of the 39.85 ppm gas concentration. For isoprene, 8 of the 120 data points were outside the control limits; however, the data displayed a random order, indicating there was no pattern to these outliers (data not shown). Similarly, for 1,3-butadiene, 8 of the 120 data points were outside the LCL and UCL. In this instance, however, a cluster of data points outside the LCL is apparent (Fig. 5). These 8 data points were obtained on the first analytical run of day 2 measurements. The 1,3-butadiene yield decreased during run five; however, this was observed only during data analysis, so there was no opportunity to investigate; if noted at the time of measurement, it would trigger further investigation and rejection of the data set. The other runs made on day 2 (runs 2, 3 and 4) were all within the control limits. Figure 5a also shows that there was a downward trend in values over the 1st day and morning of the 2nd day of measurement, but the data stabilised for the subsequent measurements.

### Repeatability of cigarette sample measurements

To further check the repeatability of the system, three different cigarette products with varying tar yields were analysed for each of the seven vapour-phase analytes. The mean yield per cigarette (n = 30) was determined by smoking each product to the butt mark. As would be expected, the highest tar yield product, CM6 (NFDPM 14 mg/cig) produced the highest yield per cigarette for all seven analytes, followed by 3R4F (NFDPM 9.4 mg/cig) and the commercial cigarette DW (NFDPM 1.9 mg/cig) (Table 7).

**Table 7:** Mean yield of analytes by cigarette type determined by ISO smoking to butt length in accordance with ISO 4387:2000 [28] (n = 30 cigarettes per product)

Product	Acetaldehyde, μg/cig	Acetone, μg/cig	1,3-Butadiene, μg/cig	2-Butanone, μg/cig	Benzene, μg/cig	Isoprene, μg/cig	Toluene, μg/cig
DW	100.3 ± 15.3	52.6 ± 6.4	11.7 ± 1.5	16.8 ± 2.1	12.3 ± 1.8	126.2 ± 14.7	15.9 ± 2.6
3R4F	519.8 ± 32.4	270.9 ± 16.8	40.6 ± 3.9	97.1 ± 6.0	49.5 ± 2.9	424 ± 8 33.4	102.6 ± 7.4
CM6	653.4 ± 55.2	343.9 ± 22.0	72.2 ± 5.6	123.5 ± 7.4	76.3 ± 4.7	707.6 ± 65.9	143.8 ± 8.3

The measurements for the three cigarette products were analysed for repeatability (r). The RSD was calculated from the average yield of each analyte per product given in Table 5. Both analyte and product variation were analysed. Regarding product variation, 3R4F showed the lowest average RSD across the seven analytes at 7.0%, followed by CM6 at 7.1% and the commercial cigarette (DW) at 13.5%. For 3R4F and CM6, all RSD values were less than the statistically relevant limit of 10% [31] (i.e., ≤ 9.7% and ≤ 9.3%, respectively). By contrast, all RSD values were above 10% (but ≤ 16.2%) for DW. This may be because the yields of the DW data were 4–6 times lower than those of the other products, with a proportionally greater impact of noise.

In terms of analyte variation, acetone and 2-butanone had the lowest RSD at 8.2%, followed by benzene (8.8%), isoprene (9.6%), toluene (9.7%), acetaldehyde (10.0%) and 1,3-butadiene (10.1%) (Table 8). By coupling a single-channel smoke machine with PI-TOF-MS via a constant flow orifice, Pang et al. [19] recently carried out an on-line analysis of the same seven compounds in mainstream smoke from 3R4F reference cigarettes, reporting RSDs below 15% for all analytes, similar to the current values.

**Table 8:** Relative standard deviation of ISO cigarette yields

Analyte	RSD, %			Mean RSD of analyte, %
	DW	3R4F	CM6	
Acetaldehyde	15.2	6.2	8.5	10.0
Acetone	12.1	6.2	6.4	8.2
1,3-Butadiene	12.8	9.7	7.7	10.1
2-Butanone	12.3	6.2	6.0	8.2
Benzene	14.5	5.9	6.1	8.8
Isoprene	11.6	7.9	9.3	9.6
Toluene	16.2	7.2	5.8	9.7
Mean RSD of product, %	13.5	7.0	7.1	

### Puff-by-puff analysis of cigarette data

**Fig. 6:**
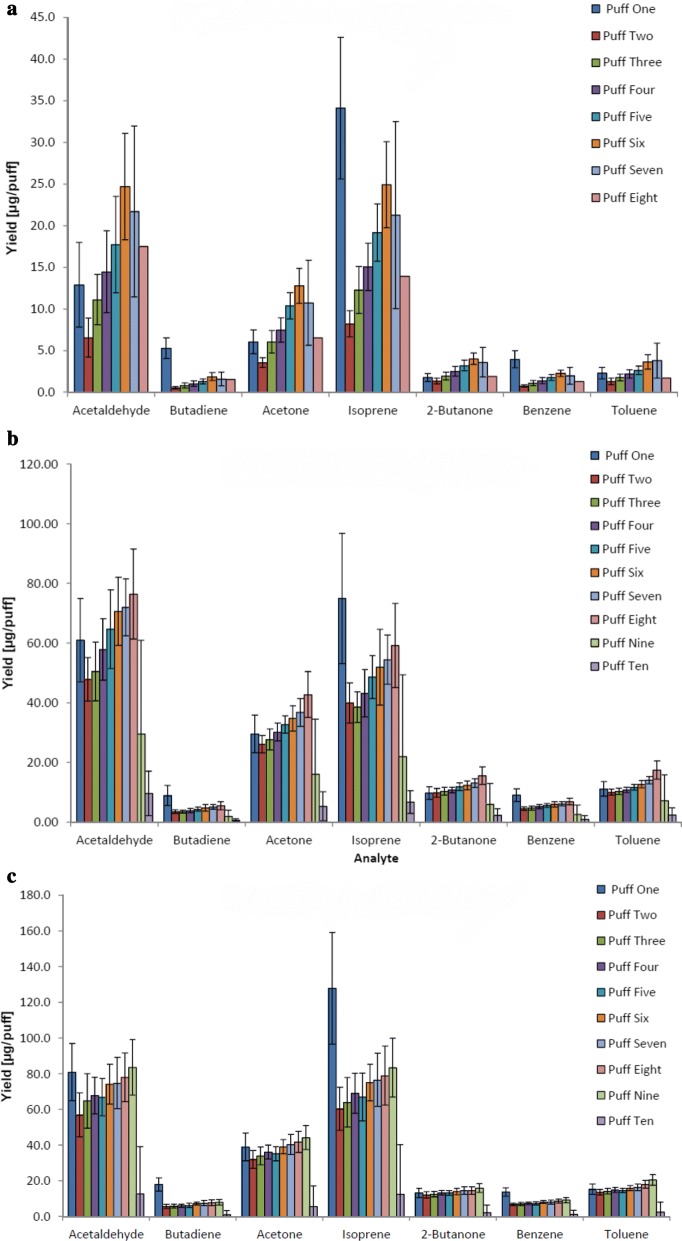
Analyte yields (Mean ± SD) on a puff-by-puff basis for each product. **a** DW. For puffs 1–6, n = 30 cigarettes; for puff 7, n = 17 cigarettes; for puff 8, n = 1 cigarette. **b** Kentucky reference 3R4F. For puffs 1–8, n = 30 cigarettes; for puff 9, n = 17 cigarettes; for puff 10, n = 2 cigarettes. **c** CORESTA monitor CM6. For puffs 1–9, n = 30 cigarettes; for puff 10, n = 15 cigarettes

The data from the LM2X-TOFMS can also be represented as yield per 35-mL puff, in keeping with the ISO smoking conditions used throughout this study. Each cigarette was smoked to the butt mark according to ISO standards (tipping paper length plus 3 mm), resulting in analyte data for up to 8–10 puffs per cigarette. Each puff was therefore compared with its counterpart in other runs. For example, all the puff-one data were averaged to obtain the mean ± SD yield for puff one (Fig. 6). Because some runs had a slightly different puff number, all graphs were normalised to the minimum consistent puff number. The number of cigarettes analysed per puff number are given in the legend.

Although the yields vary per puff, trends are apparent for most of the analytes. Apart from 2-butanone, all analytes had a visibly higher yield in the first puff than in the second puff. After the second puff, the yield increased with increasing puff number. For all three cigarette products, the first puff had the highest yield of 1,3-butadiene, isoprene and benzene. Similar puff-by-puff behaviour of analytes has been observed in previous studies [16, 20].

With increasing puff number from puff 3 to the final puff, there was an increase in mean concentration for all seven analytes for DW and 3R4F. For CM6, there was an overall increase in mean concentration with increasing puff number from puff 3, but six of the seven analytes, acetaldehyde, acetone, 2-butanone, benzene, isoprene and toluene, demonstrated a slightly lower mean for puff 5 as compared with puff 4.

For 1,3-butadiene, isoprene and benzene yields in CM6 products, puff one was unique to any other puff in the run. For CM6 products, acetaldehyde, 2-butanone and toluene exhibited the highest yield in their final puff. For 3R4F products, acetaldehyde, acetone, 2-butanone and toluene exhibited the highest yield in their final puff. For the commercial DW cigarette, only toluene exhibited the highest yield in its final puff. Notably, the large variation (i.e., SD) in the first puff indicates how different the lighting puff can be from cigarette to cigarette. This has been noted in previous studies [16], and is thought to be due to the increase in temperature in the tobacco, from room temperature to approximately 900 °C.

### Operational range of the LM2X-TOFMS and data comparison

From the certified gas mixture measurements in Table 3, a working operational range for the LM2X-TOFMS was determined. The operational range was also corrected for accuracy, as defined by the relative error reported in Table 4. The operational range and corrected operational range are summarized in Table 9.

**Table 9:** Operational range and corrected operational range

Analyte	Range, μg/puff	Corrected range, μg/puff
Acetaldehyde	8.1–81.6	11–110.9
Acetone	3.6–35.6	4.2–41.4
1,3-Butadiene	0.8–8.6	0.7–7.6
2-Butanone	2–9.5	2.2–10.3
Benzene	1.1–8.5	1.2–8.9
Isoprene	10.9–66.7	9.3–56.8
Toluene	2–13.2	2.0–13.3

The accuracy correction factors were also applied to the cigarette yield data (Table 10). The average (ISO) yield ± SD are the yields directly calculated by the LM2X-TOFMS, whereas the corrected yield ± SD are the yields that have been calculated based on the accuracy.

**Table 10:** Uncorrected and corrected ISO yields for reference 3R4F and CM6 cigarettes and commercial DW cigarette

Analyte	Product	Mean yield, μg/cig	Corrected mean yield, μg/cig
Acetaldehyde	3R4F	519.8 ± 32.4	706.4 ± 44.1
	CM6	653.4 ± 55.2	888.0 ± 75.0
	DW	100.3 ± 15.3	136.2 ± 20.8
Acetone	3R4F	270.9 ± 16.8	314.9 ± 19.5
	CM6	343.9 ± 22.0	399.8 ± 25.6
	DW	52.6 ± 6.4	61.1 ± 7.4
1,3-Butadiene	3R4F	40.6 ± 3.9	35.8 ± 3.5
	CM6	72.2 ± 5.6	63.7 ± 4.9
	DW	11.7 ± 1.5	10.3 ± 1.3
2-Butanone	3R4F	97.1 ± 6.0	105.2 ± 6.5
	CM6	123.5 ± 7.4	133.9 ± 8.1
	DW	16.8 ± 2.1	18.2 ± 2.2
Benzene	3R4F	49.5 ± 2.9	51.8 ± 3.0
	CM6	76.3 ± 4.7	79.9 ± 4.9
	DW	12.3 ± 1.8	12.9 ± 1.9
Isoprene	3R4F	424.8 ± 33.4	487.5 ± 38.4
	CM6	707.6 ± 65.9	812.2 ± 75.6
	DW	126.2 ± 14.7	144.8 ± 16.8
Toluene	3R4F	102.6 ± 7.4	103.2 ± 7.42
	CM6	143.8 ± 8.3	144.7 ± 8.37
	DW	16.0 ± 2.6	16.1 ± 2.60

**Fig. 7:**
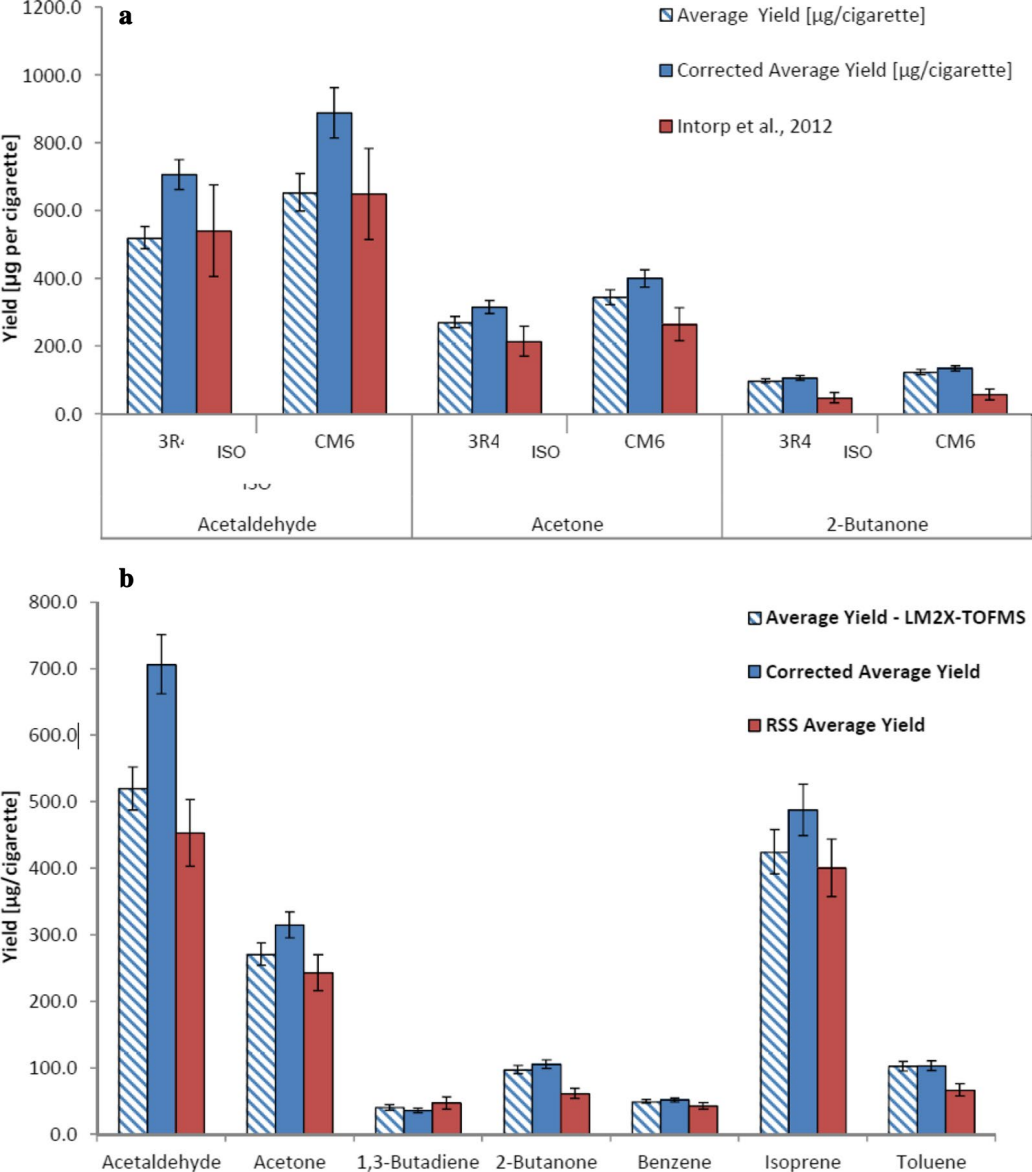
Comparison of LM2X-TOFMS smoke yields with internal and published data. **a** Selected LM2X-TOFMS cigarette yields versus external (CORESTA) data for 3R4F and CM6 carbonyl mainstream smoke yields [33]. **b** LM2X-TOFMS versus unpublished BAT data (offline methods) for 3R4F mainstream smoke yields

The corrected LM2X-TOFMS yield data were compared with internal and external published cigarette yield data. First, carbonyl measurements from the LM2X-TOFMS for 3R4F and CM6 were compared with published data generated by the CORESTA-recommended method for measuring carbonyls, involving smoke collection in impinger traps, derivatisation with 2,4-dinitrophenylhydrazine, separation of carbonyl hydrazones by reversed-phase HPLC and detection by ultra violet or diode array [33] (Fig. 7a). The 3R4F reference data, measured by the LM2X-TOFMS and corrected by accuracy (see Table 10), were then compared with comparison data generated internally by BAT (mean values per cig from 50 runs), collected by different offline methods (Fig. 7b).

Overall, the data sets compare well (Table 11). Notably, the standard deviations of the measurements performed on the LM2X-TOFMS seem to be smaller than those of the CORESTA data set [33]. The online PI-TOFMS analysis of 3R4F mainstream smoke by Pang et al. [19] also reported similar values.

**Table 11:** Comparison of 3R4F mean yield (µg/cig): real-time data (this study), real-time data [19] and offline analysis (BAT, unpublished data)

Analyte	Real-time LM2X-TOFMS (this study)	Real-time LM2X-TOFMS [19]	Offline, unpublished data (BAT)
Acetaldehyde	706.4 ± 44.1	557.0 ± 20.8	453.4
Acetone	314.9 ± 19.5	215.3 ± 10.3	242.9
1,3-Butadiene	35.8 ± 3.5	41.1 ± 5.2	47.0
2-Butanone	105.2 ± 6.5	59.8 ± 4.7	61.4
Benzene	51.8 ± 3.0	40.8 ± 3.5	42.7
Isoprene	487.5 ± 38.4	324.5 ± 24.6	400.1
Toluene	103.2 ± 7.42	69.3 ± 5.4	66.5

## Conclusion

An online mass spectrometer for puff-by-puff resolved analysis was tested and evaluated to determine its capabilities for the analysis of mainstream cigarette smoke. The LM2X-TOFMS system was found to be rugged, remaining unaffected by small changes such as changing the capillary, ferrule and/or time of day when measurements are performed. Although initial measurements indicated day-to-day variation in the measurement of some analytes, further measurements showed that day-to-day variation was not significant for all seven analytes and moving range charts showed that the system is stable and in control.

The LM2X-TOFMS demonstrated linearity across the full range of concentrations used in this study (R^2^ > 0.99 for all seven analytes). The relative error was ± 16% for six of the analytes. The largest relative error (− 36.2%) was observed for acetaldehyde, which may be due to the difference in the ionisation energy provided by the VUV lamp (ionisation source, 9.8 eV) and the first ionisation potential of acetaldehyde (10.22 eV), resulting in reduced efficiency, which is attributable to the limited overlap between the 9.8 eV of the ionisation source versus the first ionisation potential of acetaldehyde. Using an ionisation source with a higher potential might mitigate or reduce the observed variation. Further work will be required to fully understand the behaviour of acetaldehyde in the system.

Except for acetaldehyde, the analytes demonstrated good accuracy across all concentrations tested. Limits have been determined for repeatability and reproducibility that can be used for future reference. In terms of repeatability, CV(r) for the lowest gas concentration, except for isoprene, was outside the 10% guidance rules. It was difficult to assess reproducibility without another LM2X-TOFMS instrument in a different laboratory or a second operator, but limits were established for day and time of day.

Three cigarette products ranging from 2 to 14 mg of ISO tar were analysed and discriminated successfully by the instrument under the ISO regulatory smoking regime. The relative standard deviations for yields of all analytes were less than 10% for the 3R4F and CM6 products, and less than 16.2% for the lower yielding commercial cigarette. These values compare well with the accepted tolerance for measurement variability in tar, nicotine and CO under ISO smoking conditions of ± 15% for tar and nicotine, and ± 20% for CO.

Lastly, the puff-by-puff data showed that the analytes have different puff profiles. Often this was observed as a high yield in the first puff, followed by a lower yield in the second puff and then an increasing puff yield with increasing puff number. This correlates well with historic work published in this field [16, 20]. Overall, we conclude that the LM2X-TOFMS is suitable for determining the vapour-phase yields of seven analytes on a real-time, puff-by-puff basis, and can be utilised for both fast screening (qualitative) and quantitative measurements of mainstream cigarette smoke.

## Supplementary information

**Additional file 1.** Evaluation of an online, real-time, soft-photon ionisation (SPI) time-of-flight mass spectrometer for mainstream tobacco smoke analysis. **Figure S1.** Linear regression of the calculated and measured yields of acetaldehyde, acetone, benzene, 2-butanone, isoprene and toluene, demonstrating the linearity of the LM2X-TOFMS. **Table S1.** Ruggedness test plan.

## Data Availability

The datasets used and/or analysed during the current study could be available from the corresponding author on reasonable request.

## Abbreviations

CV: coefficient of variation
GC: gas chromatography
FDA: Food and Drug Administration
HCI: Health Canada Intense
HPHC: harmful and potentially harmful constituent
HPLC: high-performance liquid chromatography
MS: mass spectrometry
REMPI: resonance-enhanced multiphoton ionisation
SPI: soft photon ionisation
TOF: time of flight
VUV: vacuum ultraviolet

## References

[1] Rodgman A, Perfetti TA (2013). The chemical components of tobacco and tobacco smoke.

[2] Health Canada (2000) Tobacco reporting regulations. T-115/SOR/2000–272. http://www.wipo.int/wipolex/en/text.jsp?file_id=222850. Accessed 30 Nov 2018

[3] Food and Drug Administration (2012) Harmful and potentially harmful constituents in tobacco products and tobacco smoke: established list. US FDA. https://www.fda.gov/TobaccoProducts/Labeling/RulesRegulationsGuidance/ucm297786.htm. Accessed 30 Nov 2018

[4] Food and Drug Administration. Guidance for industry: reporting harmful and potentially harmful constituents in tobacco products and tobacco smoke under section 904(a)(3) of the Federal Food, Drug, and Cosmetic Act, March 2012. http://www.fda.gov/downloads/TobaccoProducts/GuidanceComplianceRegulatoryInformation/UCM297828.pdf. Accessed 30 Nov 2018

[5] World Health Organisation (2008). The scientific basis of tobacco product regulation.

[6] Burns DM, Dybing E, Gray N, Hecht S, Anderson C, Sanner T, O’Connor R, Djordjevic M, Dresler C, Hainaut P, Jarvis M, Opperhuizen A, Straif K (2008). Mandated lowering of toxicants in cigarette smoke: a description of the World Health Organisation TobReg proposal. Tob Control.

[7] International Organization for Standardisation (2012). Routine analytical cigarette-smoking machine—definitions and standard conditions. ISO 3308:2012.

[8] Hammond D, O’Connor RJ (2008). Constituents in tobacco and smoke emissions from Canadian cigarettes. Tob Control.

[9] Counts ME, Morton MJ, Laffoon SW, Cox RH, Lipowicz PJ (2005). Smoke composition and predicting relationships for international commercial cigarettes smoked with three machine-smoking conditions. Regul Toxicol Pharmacol.

[10] Gregg E, Hill C, Hollywood M, Kearney M, McAdam K, Purkis S, McLaughlin D, Williams M (2004). The UK smoke constituents testing study. Summary of results and comparison with other studies. Beitr Tabakforsch Intl.

[11] Australian Government (2002) Australian cigarette emissions data. Australian Government, Department of Health. http://www.health.gov.au/internet/main/publishing.nsf/content/tobacco-emis. Accessed 30 Nov 2018

[12] Borgerding MF, Bodnar JA, Wingate DE (2000) The 1999 Massachusetts benchmark study—final report. A research study conducted after consultation with the Massachusetts Department of Public Health. https://www.industrydocumentslibrary.ucsf.edu/tobacco/docs/fldf0189. Accessed 30 Nov 2018

[13] Polzin GM, Kosa-Maines RE, Ashley DL, Watson CH (2007). Analysis of volatile organic compounds in mainstream cigarette smoke. Environ Sci Technol.

[14] Sampson MM, Chambers DM, Pazo DY, Moliere F, Blount BC, Watson CH (2014). Simultaneous analysis of 22 volatile organic compounds in cigarette smoke using gas sampling bags for high-throughput solid-phase microextraction. Anal Chem.

[15] Adam T, Ferge T, Mitschke S, Streibel T, Baker RR, Zimmermann R (2005). Discrimination of three tobacco types (Burley, Virginia and Oriental) by pyrolysis single-photon ionisation–time-of-flight mass spectrometry and advanced statistical methods. Anal Bioanal Chem.

[16] Adam T, Mitschke S, Streibel T, Baker RR, Zimmermann R (2006). Puff-by-puff resolved characterisation of cigarette mainstream smoke by single photon ionisation (SPI)-time-of-flight mass spectrometry (TOFMS): comparison of the 2R4F research cigarette and pure Burley, Virginia, Oriental and Maryland tobacco cigarettes. Anal Chim Acta.

[17] Adam T, McAughey J, McGrath C, Mocker C, Zimmermann R (2009). Simultaneous on-line size and chemical analysis of gas phase and particulate phase of cigarette mainstream smoke. Anal Bioanal Chem.

[18] Mitschke S, Adam T, Streibel T, Baker RR, Zimmermann R (2005). Application of time-of-flight mass spectrometry with laser-based photo-ionisation methods for time-resolved on-line analysis of mainstream cigarette smoke. Anal Chem.

[19] Pang Y, Jiang X, Luo Y, Li X, Zhu F, Chen Z (2015). On-line puff-by-puff analysis of seven organic compounds in mainstream cigarette smoke by photo-ionisation time-of-flight mass spectrometry. Tob Sci Technol.

[20] Adam T, Mitschke S, Streibel T, Baker RR, Zimmermann R (2006). Quantitative puff-by-puff-resolved characterisation of selected toxic compounds in cigarette mainstream smoke. Chem Res Toxicol.

[21] Zimmermann R, Hertz-Schünemann R, Ehlert S, Liu C, McAdam K, Baker R, Streibel T (2015). Highly time-resolved imaging of combustion and pyrolysis product concentrations in solid fuel combustion: no formation in a burning cigarette. Anal Chem.

[22] Hertz-Schünemann R, Ehlert S, Liu C, McAdam K, Baker RR, Zimmermann R (2012). High-resolution time and spatial imaging of tobacco and its pyrolysis products during a cigarette puff by microprobe sampling photo-ionisation mass spectrometry. Anal Bioanal Chem.

[23] International Council for Harmonisation (1994). Validation of analytical procedures: text and methodology.

[24] Mühlberger F, Saraji-Bozorgzad M, Gonin M, Fuhrer K, Zimmermann R (2007). Compact ultrafast orthogonal acceleration time-of-flight mass spectrometer for on-line gas analysis by electron impact ionisation and soft single photon ionisation using an electron beam pumped rare gas excimer lamp as VUV-light source. Anal Chem.

[25] Mühlberger F, Wieser J, Ulrich A, Zimmermann R (2002). Single photon ionisation (SPI) via incoherent VUV-excimer light: robust and compact time-of-flight mass spectrometer for on-line, real-time process gas analysis. Anal Chem.

[26] International Organisation for Standardisation (1999). Tobacco and tobacco products—atmosphere for conditioning and testing. ISO 3402:1999.

[27] Eldridge A, Betson TR, Vinicius Gama M, McAdam K (2015). Variation in tobacco and mainstream smoke toxicant yields from selected commercial cigarette products. Regul Toxicol Pharm.

[28] International Organisation for Standardisation (2000). Cigarettes—determination of total and nicotine-free dry particulate matter using a routine analytical smoking machine. ISO 4387:2000.

[29] International Organisation for Standardisation (1994). Accuracy (trueness and precision) of measurement methods and results—part 2: basic method for the determination of repeatability and reproducibility of a standard measurement method. ISO 5725-2:1994.

[30] Wheeler DJ, Chambers DS (1992). Understanding statistical process control.

[31] Automotive Industry Action Group (2010). Measurement systems analysis.

[32] Horwitz W, Kamps LR, Boyer KW (1980). Quality assurance in the analysis of foods for trace constituents. J Assoc Off Anal Chem.

[33] Intorp M, Purkis S, Wagstaff W (2012). Determination of carbonyl compounds in cigarette mainstream smoke, The CORESTA 2010 collaborative study and recommended method. Beit Tabakforsch Intl.

