# Supplementary material for: Evaluation of an online, real-time, soft-photon ionisation time-of-flight mass spectrometer for mainstream tobacco smoke analysis
Source: BMC Chem. 2019 Dec 21;13(1):135. doi: 10.1186/s13065-019-0654-z (PMC6925416; doi:10.1186/s13065-019-0654-z)
Supplement: Supplementary file 1 — Additional file 1. Evaluation of an online, real-time, soft-photon ionisation (SPI) time-of-flight mass spectrometer for mainstream tobacco smoke analysis. Figure S1. Linear regression of the calculated and measured yields of acetaldehyde, acetone, benzene, 2-butanone, isoprene and toluene, demonstrating the linearity of the LM2X-TOFMS. Table S1. Ruggedness test plan. [file 13065_2019_654_MOESM1_ESM.docx]

**Evaluation of an online, real-time, soft-photon ionisation (SPI) time-of-flight mass spectrometer for mainstream tobacco smoke analysis – Supplementary Figures**

Jenni Hawke^1^, Graham Errington^1^, John McAughey^1^, and Matthias Bente von Frowein^2^

**Figure S1**


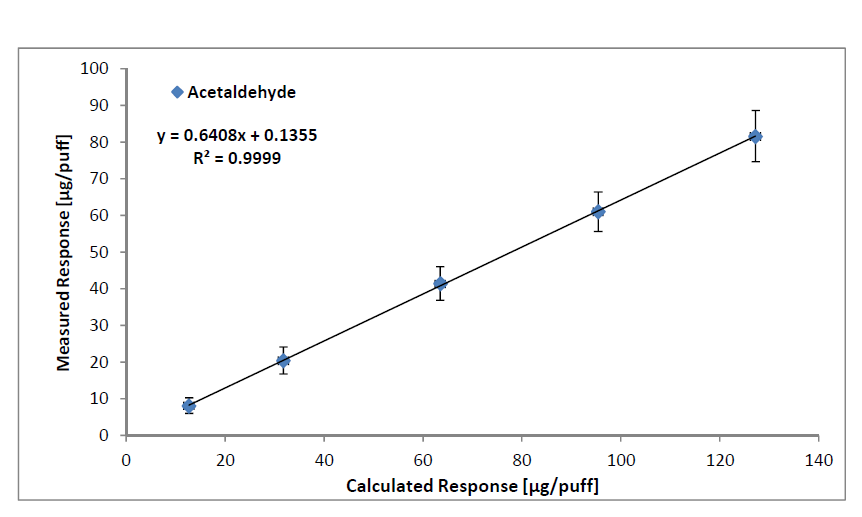

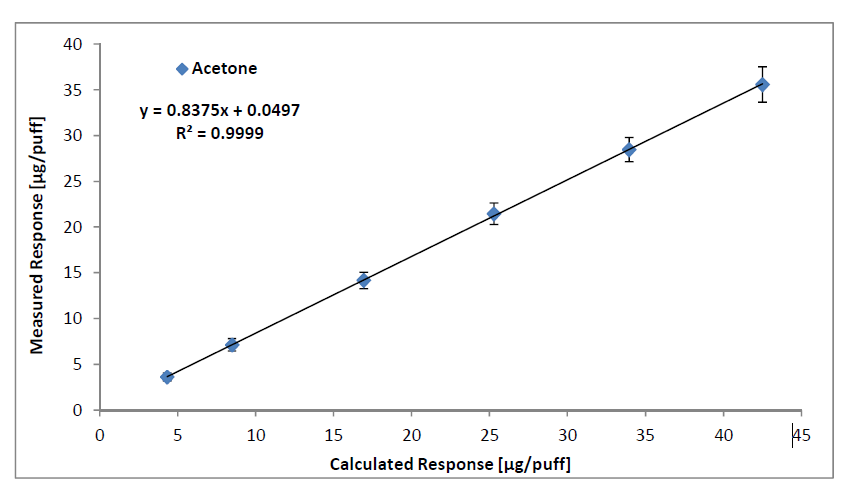


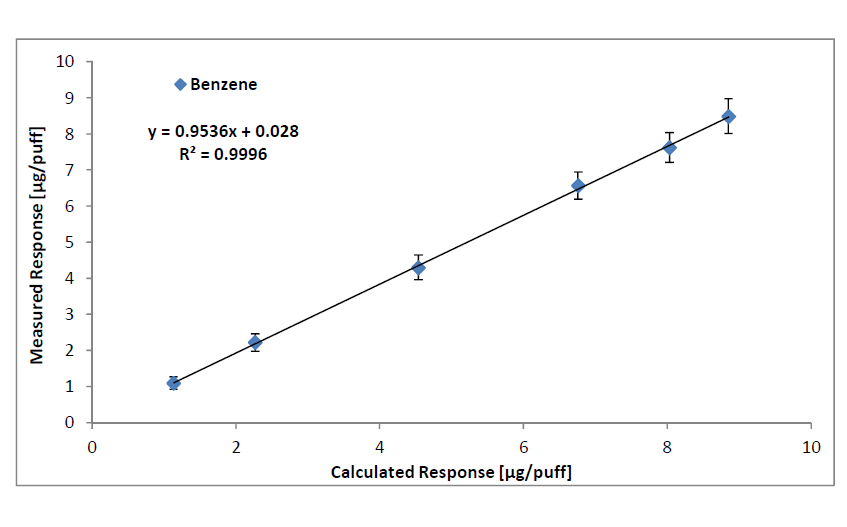

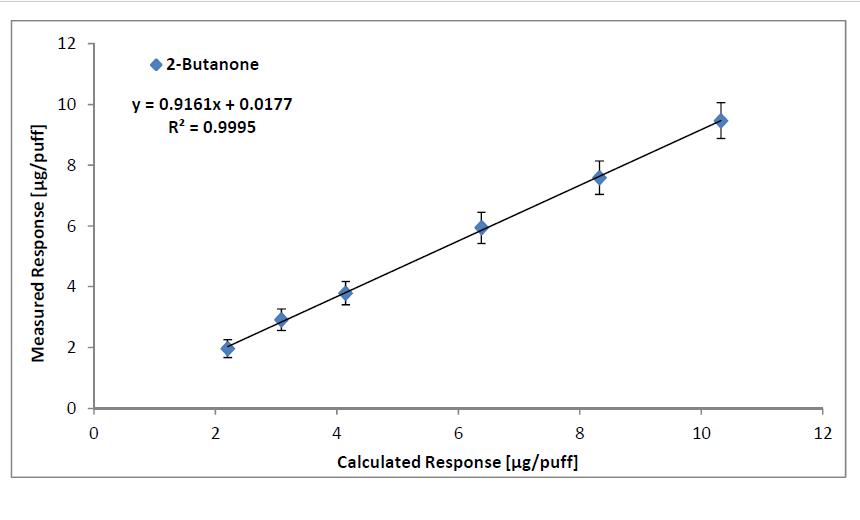


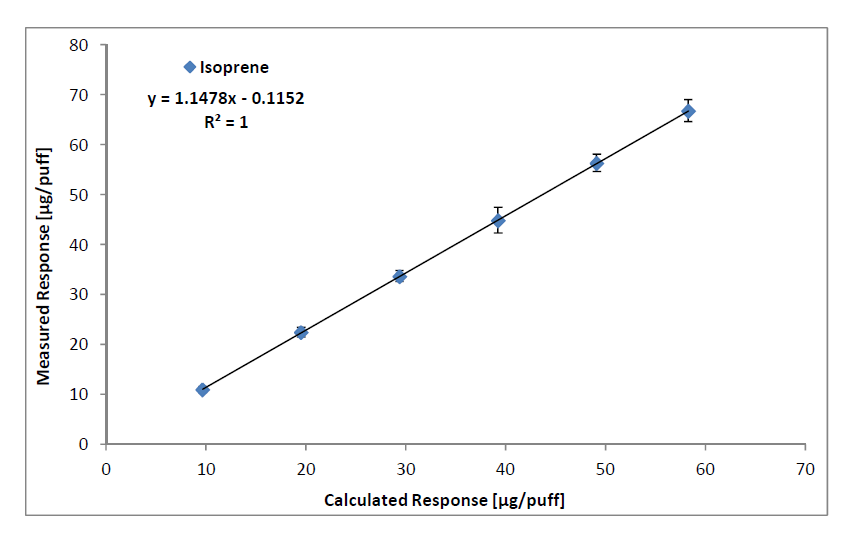

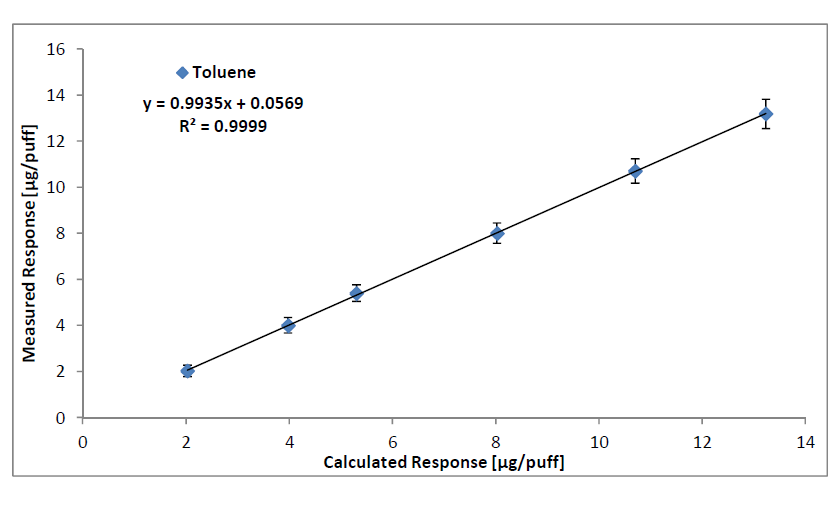


**Figure S1**. Linear regression of the calculated and measured yields of acetaldehyde, acetone, benzene, 2-butanone, isoprene and toluene, demonstrating the linearity of the LM2X-TOFMS.

**Table S1**

| **Table S1.** Ruggedness test plan | | | | | |
| --- | --- | --- | --- | --- | --- |
| **Trial** | **Capillary** | **Ferrule** | **Day** | **Time** | **Run** |
| Day 1 |  |  |  |  |  |
| 1 | New | New | 1 | am | 1 |
| 1 | Cut 1 | Same | 1 | am | 2 |
| 1 | Cut 2 | Same | 1 | am | 3 |
| 1 | Cut 3 | Same | 1 | am | 4 |
| 1 | No cut | Same | 1 | am | 5 |
| 2 | New | New | 1 | pm | 6 |
| 2 | Cut 1 | Same | 1 | pm | 7 |
| 2 | Cut 2 | Same | 1 | pm | 8 |
| 2 | Cut 3 | Same | 1 | pm | 9 |
| 2 | No cut | Same | 1 | pm | 10 |
| Day 2 |  |  |  |  |  |
| 3 | New | New | 2 | am | 1 |
| 3 | Cut 1 | Same | 2 | am | 2 |
| 3 | Cut 2 | Same | 2 | am | 3 |
| 3 | Cut 3 | Same | 2 | am | 4 |
| 3 | No­­ cut | Same | 2 | am | 5 |
| 4 | New | New | 2 | pm | 6 |
| 4 | Cut 1 | Same | 2 | pm | 7 |
| 4 | Cut 2 | Same | 2 | pm | 8 |
| 4 | Cut 3 | Same | 2 | pm | 9 |
| 4 | No­ Cut | Same | 2 | pm | 10 |
| Day 3 |  |  |  |  |  |
| 5 | New | New | 3 | am | 1 |
| 5 | Cut 1 | Same | 3 | am | 2 |
| 5 | Cut 2 | Same | 3 | am | 3 |
| 5 | Cut 3 | Same | 3 | am | 4 |
| 5 | No cut | Same | 3 | am | 5 |
| 6 | New | New | 3 | pm | 6 |
| 6 | Cut 1 | Same | 3 | pm | 7 |
| 6 | Cut 2 | Same | 3 | pm | 8 |
| 6 | Cut 3 | Same | 3 | pm | 9 |
| 6 | No cut | Same | 3 | pm | 10 |
